# From the Eukaryotic Molybdenum Cofactor Biosynthesis to the Moonlighting Enzyme mARC

**DOI:** 10.3390/molecules23123287

**Published:** 2018-12-11

**Authors:** Manuel Tejada-Jimenez, Alejandro Chamizo-Ampudia, Victoria Calatrava, Aurora Galvan, Emilio Fernandez, Angel Llamas

**Affiliations:** Departamento de Bioquímica y Biología Molecular, Campus de Rabanales y Campus Internacional de Excelencia Agroalimentario (CeiA3), Edif. Severo Ochoa, Universidad de Córdoba, 14071 Cordoba, Spain; q62tejim@uco.es (M.T.-J.); alejandro.chamizo@unavarra.es (A.C.-A.); calatravavictoria@gmail.com (V.C.); bb1gacea@uco.es (A.G.); bb1feree@uco.es (E.F.)

**Keywords:** molybdenum cofactor, mARC, moonlighting, nitrite, nitric oxide, cytochrome b5, nitrate reductase, NOFNiR, MOSC

## Abstract

All eukaryotic molybdenum (Mo) enzymes contain in their active site a Mo Cofactor (Moco), which is formed by a tricyclic pyranopterin with a dithiolene chelating the Mo atom. Here, the eukaryotic Moco biosynthetic pathway and the eukaryotic Moco enzymes are overviewed, including nitrate reductase (NR), sulfite oxidase, xanthine oxidoreductase, aldehyde oxidase, and the last one discovered, the moonlighting enzyme mitochondrial Amidoxime Reducing Component (mARC). The mARC enzymes catalyze the reduction of hydroxylated compounds, mostly N-hydroxylated (NHC), but as well of nitrite to nitric oxide, a second messenger. mARC shows a broad spectrum of NHC as substrates, some are prodrugs containing an amidoxime structure, some are mutagens, such as 6-hydroxylaminepurine and some others, which most probably will be discovered soon. Interestingly, all known mARC need the reducing power supplied by different partners. For the NHC reduction, mARC uses cytochrome b5 and cytochrome b5 reductase, however for the nitrite reduction, plant mARC uses NR. Despite the functional importance of mARC enzymatic reactions, the structural mechanism of its Moco-mediated catalysis is starting to be revealed. We propose and compare the mARC catalytic mechanism of nitrite versus NHC reduction. By using the recently resolved structure of a prokaryotic MOSC enzyme, from the mARC protein family, we have modeled an in silico three-dimensional structure of a eukaryotic homologue.

## 1. Biological Relevance of Molybdenum Cofactor

**Figure 1:**
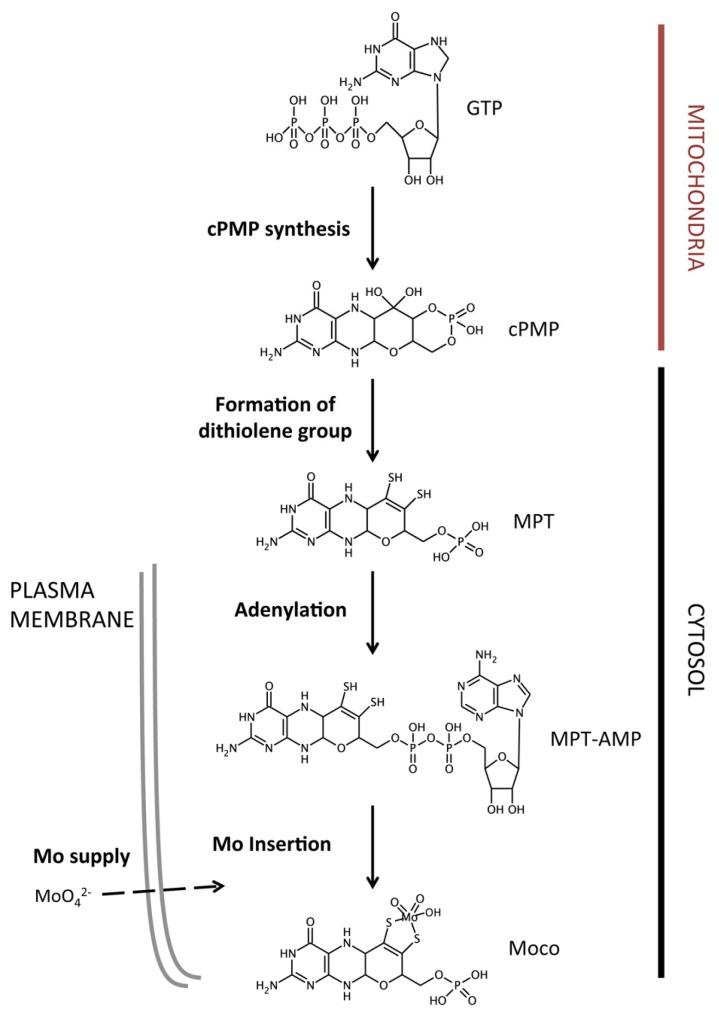
Schematic representation of the Moco biosynthetic pathway. Metabolic steps involved in Moco biosynthesis, together with the main intermediates are shown. Subcellular localization of each step is shown on the right side. GTP, guanosine triphosphate; cPMP, cyclic pyranopterin monophosphate; MTP, molybdopterin; MTP-AMP, adenylated molybdopterin.

Among the transition metals of the fifth row of the periodic table, Mo is the only one that has been reported as an essential element. In 1939, Arnon and Stout reported for the first time its importance in biology using tomato plants growing in a defined nutrient solution [1]. Under Mo deficiency conditions, these plants showed an aberrant development that disappeared when Mo was added to the nutrient solution. Later on, Mo was found to be a cofactor for more than fifty enzymes in organisms belonging to all kingdoms of life [2]. However, Mo is not biologically active by itself, but in form of a pterin-derived cofactor: the Moco (Figure 1), constituted by a reduced pterin with a side chain making a pyran ring containing a dithiolene and phosphate groups [2].

All eukaryotes, except for some unicellular eukaryotes, such as parasites and some yeasts, utilize Mo [3]. In eukaryotes, Mo is present in the active center of five enzymes that are responsible for important steps in nitrogen, sulphur, and carbon metabolism, such as nitrate reductase (NR), sulphite oxidase (SO), aldehyde oxidase (AO), xanthine oxidoreductase/dehydrogenase (XOR/XD), and mitochondrial amidoxime reducing component (mARC) [4]. Mo-related deficiencies lead to the pleiotropic loss of the activity of all Moco enzymes. It can be caused either by an insufficient Mo supply or by a defect in Moco biosynthesis. In plants, Mo-deficiency leads to impaired plant development, severely impacting the overall plant growth [5]. This phenotype is mainly caused by the loss of NR and AO activities, and is responsible for nitrate reduction as a first step of nitrate assimilation and the phyto-hormone abscisic acid (ABA) biosynthesis, respectively [6,7]. ABA has been reported as an important player in plant development and stress adaptation [8]. In mammals, Moco-deficiency causes a severe neurological disorder that leads to death shortly after birth, mainly due to the SO activity deficiency, resulting in sulphite accumulation and subsequent brain damage [9]. However, problems that are related to Mo-derived toxicity are very rare and have been described mainly in ruminants. In these organisms, high Mo intake leads to molybdenosis, which is characterized by the formation of tetrathiomolybdate that inhibits copper trafficking proteins, resulting in copper-deficiency in these animals [10].

## 2. The Molybdenum Cofactor Biosynthetic Pathway

In eukaryotes, Moco is synthesized by a highly-conserved metabolic pathway starting from 5’-GTP and comprising four biosynthetic steps that take place within the mitochondria and cytosol (Figure 1). At least six genes are needed for Moco biosynthesis in eukaryotes. The nomenclature that is used for these genes varies among the organisms; while human genes use the MOCS nomenclature (MO Cofactor Synthesis), plants follow the CNX nomenclature (Cofactor for Nitrate reductase and Xanthine dehydrogenase) [11]. In addition, Moco synthesis requires Mo supply into the cell in the form of the oxyanion molybdate (MoO_4_^2−^), being mediated by specific molybdate transporters [12].

### 2.1. Molybdate Transport

In order to synthesize Moco, all known living organisms need to acquire Mo from the external medium in form of molybdate. In eukaryotes, transporters mediating specific and high-affinity molybdate transport belong to the MOT1 or MOT2 families [4,13]. The first eukaryotic transporter identified as being involved in molybdate transport belongs to the MOT1 family (MOlybdate Transporter type 1) and it was identified simultaneously in the green alga *Chlamydomonas reinhardtii* and in the plant *Arabidopsis thaliana* [14,15]. Proteins showing high similarity to MOT1 are present in bacteria, fungi, algae, and higher plants, and they share two sequence motifs that are likely involved in molybdate transport [14]. In *C. reinhardtii* MOT1 (CrMOT1) seems to be related to Mo supply for NR activity. CrMOT1 transcription and activity are regulated by the presence of nitrate as nitrogen source, but are unaffected by Mo availability [14]. This effect may be explained by the requirement of the coordination between nitrate assimilation and Mo homeostasis due to the Moco requirement for an appropriate NR activity. In *A. thaliana,* two members of the MOT1 family (AtMOT1 and AtMOT2) have been reported to be involved in Mo transport [15,16]. AtMOT1 is a high-affinity and specific molybdate transporter that is crucial for efficient Mo uptake from the soil [15]; however, its physiological role still needs to be clarified since it has been reported in two different subcellular localizations: plasma membrane and mitochondria [15,17]. AtMOT2 is a vacuolar molybdate transporter that is most likely involved in Mo storage and Mo homeostasis in the cytosol [16]. Members of the MOT1 family have also an important role in Mo homeostasis in legume plants, particularly connected to symbiotic nitrogen fixation (SNF) [18,19]. In fact, Mo is an essential metal for SNF and it is required for the activity of the central enzyme of this process, nitrogenase [20]. In *Medicago truncatula*, MtMOT1.3 and MtMOT1.2 are responsible for Mo supply to the nitrogen-fixing tissues. While MtMOT1.2 mediates Mo that is released by the vasculature to the organs where the nitrogen fixation takes places, the nodules [21]; MtMOT1.3 is responsible for Mo transport into nodule cells from the nodule apoplast [19].

A second family of eukaryotic specific molybdate transporters was identified in *C. reinhardtii* (MOT2) [22]. CrMOT2 shares only about 12% identity with MOT1 proteins, and it is present not only in algae and plants, but also in animals, including humans. Indeed, heterologous expression in yeast suggested that human MOT2 also mediates molybdate transport, being the first human protein that has been related to Mo homeostasis [22]. However, since the MOT2 proteins function as molybdate transporter has only been verified in *C. reinhardtii*, more experiments are needed to confirm this role in other eukaryotes.

### 2.2. Molybdopterin Synthesis

Moco biosynthesis starts with molybdopterin (MPT) synthesis, which in turn comprises two different metabolic steps: cPMP and dithiolene synthesis (Figure 1). The first step entails the conversion of 5’-GTP into cyclic pyranopterin monophosphate (cPMP) within the mitochondrial matrix [23]. cPMP consists in a sulfur-free pyranopterin containing an uncommon geminal diol group, being the most stable intermediate within the Moco biosynthetic pathway [24,25]. In plants, this reaction requires two gene products, CNX2 and CNX3; while in humans, it is carried out by MOCS1A and the multidomain protein MOCS1B, both encoded by the MOCS1 gene as the result of several splice variants [26,27]. Once cPMP is synthesized, it is exported to the cytosol in a process that is mediated by the mitochondrial inner membrane transporter ATM3 [28]. In the cytosol, two sulfur atoms are incorporated to the cPMP molecule, in the so-called dithiolene reaction step, to form MPT. This reaction is mediated by the MTP synthase complex, which is formed by two monomers of CNX6/MOSC2B (large subunit) and two monomers of CNX7/MOSC2A (small subunit) [29]. While in plants CNX6 and CNX7 are encoded by independent genes, in humans MOSC2A and MOSC2B are encoded by the bicistronic gene MOSC2 [30]. After dithiolene reaction, MPT synthase requires resulfuration that is mediated by the proteins CNX5 and MOSC3 in plants and humans, respectively [31].

### 2.3. Molybdenum Insertion into Molybdopterin

The last step of Moco synthesis implies Mo insertion into MPT to yield the active cofactor. However, this insertion cannot take place immediately after MPT synthesis, since this molecule requires a prior activation consisting in the MPT adenylation. In plants, MTP adenylation is mediated by CNX1, but in humans, it is Gephyrin. These proteins contain two domains (G and E domains), that are involved not only in adenylation, but also in the final Mo insertion. Interestingly, *A. thaliana* CNX1 and Gephyrin show an opposite domain orientation. In *C. reinhardtii*, the CNX1G and CNX1E domains are encoded by independent genes and chimeric fusions containing these two domains with different orientations are able to synthesize Moco [32]. The three-dimensional structure of *A. thaliana* CNX1G revealed that the MPT adenylation reaction is Mg^2+^- and ATP-dependent and it leads to MPT-AMP that is bound to CNX1G domain [33]. Subsequently, MPT-AMP is transferred to the E-domain where the deadenylation reaction takes place in a Zn^2+^/Mn^2+^ dependent manner [34]. Molybdate also binds to the E-domain triggering the AMP hydrolysis and yielding the active Moco. Arabidopsis CNX1 is able to bind to the cytoskeleton, where it interacts with other proteins that are involved in Moco biosynthesis, pointing to CNX1 as anchor protein for this metabolic pathway [35,36].

### 2.4. Molybdenum Cofactor Insertion

**Figure 2:**
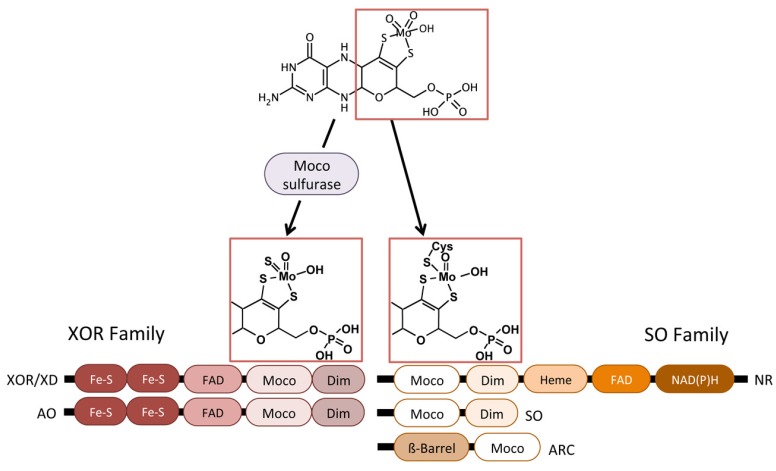
Eukaryotic Moco-containing enzymes. Domain structures of eukaryotic Mo-enzymes of the xanthine oxidoreductase and sulfite oxidase families. Moco final maturation needed for its transference to the corresponding Mo-enzyme family is shown. XOR/XD, xanthine oxidoreductase/xanthine dehydrogenase; AO, aldehyde oxidase; NR, nitrate reductase; SO, sulfite oxidase; mARC, mitochondrial amidoxime reducing component; Dim, dimerization domain.

Once eukaryotic Moco is synthesized, it can bind directly to SO, NR, or mARC apoenzymes, or it can undertake a final sulfuration in order to bind to the apoenzymes AO or XOR/XD (Figure 2). This sulfuration process is catalyzed by the Moco sulfurase enzyme, ABA3 in plants, and MCSU in humans [37,38]. In *A. thaliana* ABA3, the N-terminal domain (NifS-like domain) mediates the desulfuration of l-cysteine forming a persulfide intermediate on a conserved cysteine residue, which is subsequently used by the C-terminal domain and it is responsible for Moco sulfuration [39,40].

The free Moco is highly sensitive to oxidation. In vitro, it exhibits a half-life of only a few minutes [41]. Thus, eukaryotic cellular systems seem to have developed mechanisms for Moco protection and storage that are achieved by means of Moco binding proteins (MCP) [41,42,43]. In *C. reinhardtii*, MCP is able to bind and protect Moco, increasing its half-life up to two days and is also involved in the transference of this cofactor to apo-enzymes [41,44]. *C. reinhardtii* MCP works as a homotetrameric protein being each monomer 16.5 kDa size. The MCP crystal structure shows a Rossman fold that is present in each monomer and the putative Moco-binding site has been proposed [42]. In addition, in silico docking studies together with a mutagenesis strategy suggested a putative Moco binding site. Mutations in conserved residues within this putative binding site result in a reduced capacity to bind or protect Moco [42]. No MCP homologues have been found in higher plant genomes; however, members of a family of lysine decarboxylase-like proteins (MoBP), sharing structural similarity with MCP, are able to bind Moco or MPT in *A. thaliana* [43]. MoBPs are able to interact with CNX1 and the Mo-containing enzyme, NR. Also, they promote CNX1-ABA3 interaction, followed by ABA3-XOR/AO interaction, probably to insert the cofactor into the corresponding apo-enzyme [36,43].

## 3. Eukaryotic Molybdenum Cofactor Enzymes

The biological activities of more than fifty enzymes rely on Mo. Among them, five are present in eukaryotes, NR, SO, AO, XOR/XD, and mARC [4,45]. These eukaryotic enzymes mediate redox reactions and they are classified into two groups, the SO family and the XOR family, according to the ligands that bind the cofactor to the active center (Figure 2) [46]. The SO family comprises NR, SO and mARC, these enzymes carry the cofactor with two oxido- and a cysteine ligands, while the XOR includes the AO and XOR/XH enzymes with the cofactor containing a sulfido- and oxido-ligands [46].

Among the eukaryotic Mo-containing enzymes, NR is the only one that is not present in animals. NR is functional as a homodimer, each monomer contains a FAD, Heme b557, and Moco together with a NAD(P)H-binding and dimerization sites. This enzyme catalyzes the reduction of nitrate to nitrite as a key step of the nitrate assimilation pathway [45]. Also, NR has been related to nitric oxide (NO) homeostasis, since, using its diaphorase partial activity, it can either supply electrons to the mARC enzyme to reduce nitrite to NO, or transfer these electrons to the Truncated Hemoglobin 1 (THB1) to convert NO to nitrate [47,48].

The SO enzyme acts as homodimeric protein and participates in sulfur catabolism mediating the two-electron oxidation of sulfite to sulfate [49]. In vertebrates, SO transfers the electrons to cytochrome c, and each monomer contains a heme, Moco, and dimerization domain [50]. Furthermore, animal SO is localized in the mitochondrial intermembrane space and it carries out sulfite oxidation as final step of cysteine degradation [51]. In plants, SO seems to be involved in plant protection from sulfite toxicity under SO_2_ rich atmosphere [52]. The plant enzyme uses O_2_ as electron acceptor producing H_2_O_2_ as side product and it is localized in the peroxisome [53,54]. Furthermore, plant SO lacks the heme domain that is present in animals [54], however the unicellular alga *C. reinhardtii* shows a SO with cytochrome b5 similar to vertebrates [55].

XOR/XD enzymes are involved in purine degradation by mediating the oxidation of hypoxanthine to xanthine and xanthine to uric acid. They contain 2Fe-2S, FAD, Moco, and dimerization domains, resulting in a 150 kDa protein that undergoes dimerization to gain biological activity [46]. These prosthetic groups participate in electron transfer from the substrate to NAD+ (due to its dehydrogenase activity) or to O_2_ (due to its oxidase activity) to form NADH or superoxide respectively [56]. In addition, plant XD shows a NADH oxidase activity yielding NAD+ and superoxide [57]. It has been proposed that plant XD could participate in reactive oxygen species metabolism by playing two parallel tasks, on one hand it can contribute to pathogen resistance by H_2_O_2_ generation, and on the other hand this enzyme produces uric acid and removes H_2_O_2_ from chloroplasts under oxidative stress [58].

AO proteins share a high similarity in sequence and structure with XOR/XD, since in eukaryotes the AO gene was originated from a duplication of XDH gene [59]. They also have the same domains for cofactor binding and share similar reaction mechanism [60]. In general, AO shows wide substrate specificity, including aldehydes, purines, pteridines, and heterocycles with O_2_ as terminal electrons acceptor and generating H_2_O_2_ [61]. In plants, AO is involved in ABA biosynthesis by abscisic aldehyde oxidation [62]. ABA acts as phytohormone and it is connected to the signaling of biotic and abiotic stress. The physiological role of animal AO is still unknown, although it could participate in oxidation of a broad variety of endogenous substrates, such as vitamins of neurotransmitters [61].

## 4. mARC More Than Just “One Enzyme”

The mARC is the last Moco enzyme discovered in eukaryotic organisms. The first evidence for this finding was in bacteria, where it was observed that a defect in Moco biosynthesis led to a hypersensitive phenotype to N-hydroxylated (NHC), the base analogue of 6-hydroxylaminopurine (HAP), a powerful mutagen for bacteria and eukaryotic cells [63]. However, none of the Moco enzymes that were known until then were responsible for this phenotype. The identity of the enzyme was deciphered when studies on the reduction of amidoximes by liver mitochondria revealed a novel Moco-dependent enzymatic activity, involved in the reduction of the NHC prodrug benzamidoxime to its active form benzamidine [64]. Therefore, the name that was chosen for this new Moco enzyme was mARC (mitochondrial Amidoxime Reducing Component). Since then, a great variety of other substrates, apart from amidoximes, have been assigned to mARC.

**Figure 3:**
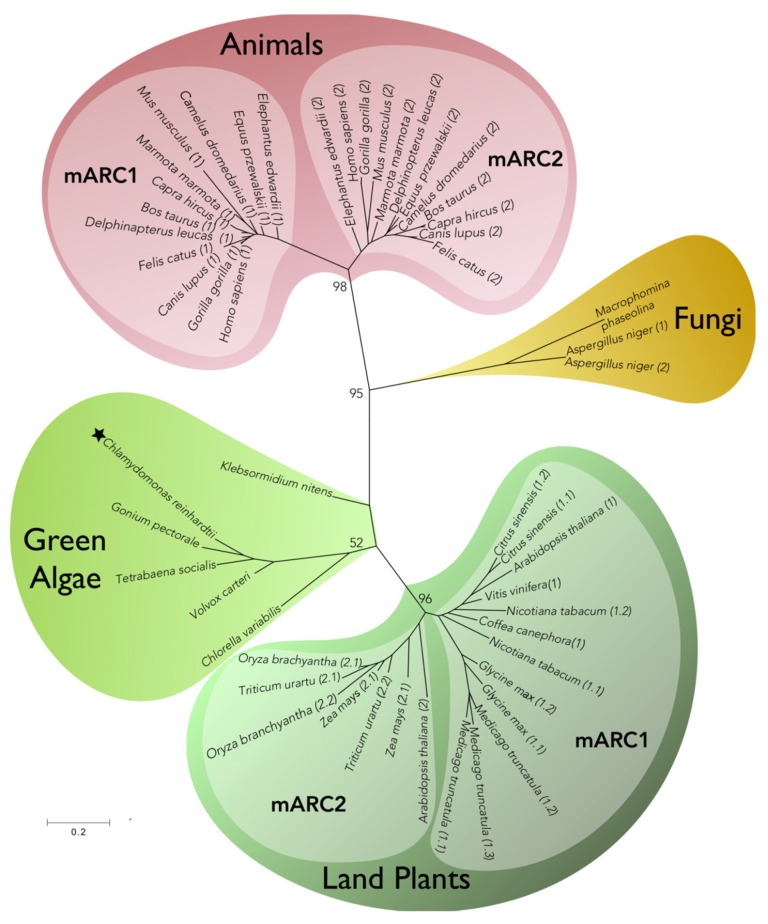
Phylogenetic relationships of putative eukaryotic mARC enzymes. The tree was constructed using the Maximum Likelihood method integrated in the software MEGA7 [80]. The alignment was obtained using Clustal method, and the evolutionary distances were computed using the Poisson correction method. Numbers in branches show bootstrap values (%). The distances are units of the number of amino acid substitutions per site. Animal and plant members are named as (1) or (2) corresponding to Homo sapiens and *Arabidopsis thaliana* mARC1 and mARC2 homologs, respectively. NCBI proteins ID: (black star) *C. reinhardtii: AEI61922.1; T. socialis: PNH06437.1; C. variabilis: XP_005847212.1; G. pectorale: KXZ54122.1; V. carteri: XP_002954725.1; K. nitens: GAQ87885.1; M. phaseolina: EKG10021.1; A. niger (1): CAK45930.1, (2): SPB51236.1; N. tabacum (1.1): XP_016495235.1, (1.2): XP_016443939.1; C. sinensis (1.1) XP_006468577.1, (1.2) XP_006468578.1; M. truncatula: (1.1) XP_003594861.1, (1.2): XP_003594859.2, (1.3): XP_003594858.1; Z. mays (2.1): NP_001148545.1, (2.2): NP_001348577.1; G. max (1.1): XP_014617397.1, (1.2): XP_003533626.2; V. vinifera (1): XP_002273557.1; C. canephora (1): CDP18170.1; O. branchyantha (2.1): XP_006660977.1; (2.2): XP_006661538.1; T. urartu (2.1): EMS64933.1, (2.2): EMS64934.1. A. thaliana (1): NP_174376.1, (2): NP_199285.1; B. taurus (1): XP_002694007.2, (2): NP_001069848.1; H. sapiens (1): NP_073583.3, (2): NP_060368.2; C. dromedarius (1): XP_010991227.1, (2) XP_010991233.1; M. marmota (1): XP_015339107.1, (2): XP_015339108.1; C. lupus (1): XP_005640886.2, (2): XP_005640884.1; F. catus (1): XP_023103250.1, (2): XP_023102999.1; M. musculus (1): NP_001277202.1, (2): NP_598445.1; E. edwardii (1): XP_006894605.1, (2) XP_006894606.1; G. gorilla (1): XP_018891553.1, (2): XP_018886259.1; D. leucas (1): XP_022434969.1, (2): XP_022434968.1; C. hircus (1): XP_017915667.1, (2): XP_017915666.1; E. przewalskii (1): XP_008527451.1, (2): XP_008527452.1.*

The presence of genes encoding mARC seems to be a general feature in eukaryotic organisms. Genomes from animals, fungi, plants, and algae were analyzed to determinate the number of mARC homologs in different species and to build a phylogenetic tree. As shown in Figure 3, one or two genes encoding for putative mARC were identified in eukaryotic genomes, with the exception of *M. truncatula*, which contains three members. Within the animal branch, it is observed that all species contain two mARC members, each one fitting into two different sub-branches. In contrast, within the plant branch, mARCs from each plant species fit into a single sub-branch, except for *A. thaliana*. In the green algae branch, where *C. reinhardtii* (Chlorophyta) and the closely related to the plant ancestor *Klebsormidium* (Charophyta) are included, single mARC homologs were identified in each genome.

Two characteristics could be assigned to the eukaryotic mARC enzymes, the Zn^2+^ requirement and the ability to interact with other proteins. The Zn^2+^ requirement was shown in *C. reinhardtii* where the enzymatic activity needs Zn^2+^ [65], but also from a proteomic study where the Zn^2+^ deficiency increases the cellular crARC content more than 30 times [66]. The recombinant expression of human mARC proteins in *E. coli* revealed that these enzymes are mainly monomeric, in contrast to all other eukaryotic Moco enzymes, which are homo- or hetero-dimers [67]. The mARC monomer is about 30 kDa and it belongs to the SO family, since an absolutely conserved cysteine has been identified as the fifth Mo ligand [65]. However, the human mARC [68] and crARC [69] seem to form in certain conditions a high molecular oligomeric complex of more than 350 kDa in size (10–12 monomers). Generally, eukaryotic mARC enzymes contain two domains, a MOSC domain (MOS C-terminal domain) that is present in the MOS (Moco sulfurases) enzymes and a β-barrel domain [70]. The MOS enzymes are involved in the transference of a sulfide ligand to Moco, and the resulting sulfurated Moco is essential for the activity of XOR/XD family enzymes [37]. The MOSC domain seems to be involved in Moco binding [71]. The N-terminal β-barrel domain may have specific roles in the interaction with the substrates of these enzymes and it is predicted to form a β-strand-rich fold-like structure [70,72]. The amino acids R276, C252, and F210 were found to be critical for Moco chelation in crARC, while D182 was important for the crARC reduction activity [65,69].

A fascinating aspect about mARC enzymes is that they require protein partners providing electrons from NADH to reduce the substrate. Therefore, the name ARCO (Amidoxime Reducing Complex) has been proposed for the enzymatic complex formed between mARC and its partners. Interestingly, the type of partner varies depending on which enzymatic activity will be developed. According to this, mARC enzymes have been proposed to be moonlighting enzymes [73]. The moonlighting proteins form a special class of multifunctional enzymes that are able to perform different physiological functions depending on the concentration of cellular ligands, substrates, cofactors, tissue, cellular localization, oligomeric state, or post-translational modifications [74]. The β-barrel domain, which is predicted in mARC, has been found to be the most frequent fold between moonlighting proteins [75]. In agreement with its moonlighting behavior, mARC has been identified in different subcellular compartments. The human mARCs has been localized in the mitochondrial membrane [76] with its C-terminus catalytic domain exposed to the cytosol [68], which is consistent with the localization and the orientation of its partners, Cytb5 and Cytb5-R [77]. However, rat mARC2 is located in the peroxisomal membranes [78] and mouse mARCs proteins in the inner mitochondria membrane [79]. Interestingly, Arabidopsis mARCs lack clear targeting signals for organelle localization [11], and the Chlamydomonas crARC is located in the cytosol, as is consistent with the localization of its partner [47]. According to its moonlighting behavior, mARC proteins have been found to be involved in the reduction of a wide range of substrates in different metabolic pathways, which will be analyzed in the following sections.

## 5. The NHC Reduction Capacity of mARC

The NHC reduction activity that is mediated by eukaryotic mARC needs two additional protein partners, Cytb5 and Cytb5-R [64]. However, in *E. coli,* the mARC homolog (YcbX) contains a ferredoxin domain fused to its C-terminus domain [81] and needs only a protein partner, the CysJ protein that is also a component of the sulfite reductase complex [82]. Briefly, the ARCO activity is the result of an electron flux chain that provides the reducing power that is needed for NHC reduction. However, bacterial ARCO is a two-component system where the electrons from NADH are funneled from CysJ to YcbX. In contrast, eukaryotic ARCO is a three-component system where the electrons are funneled from Cytb5-R and Cytb5 to mARC [65].

Several mARC proteins have been involved in the reduction of N-hydroxylated nucleobases and nucleosides (N-hydroxycytosine, HAP, N-hydroxycytidine, and N-hydroxyadenosine), which are potent mutagenic and toxic compounds [83]. Consistent with this, the RNAi mediated down-regulation of human mARCs led to the decrease of N-reductive detoxification of HAP [84]. The plant [65] and bacterial mARC [85] are involved in the detoxification of HAP to adenine. Some mARC enzymes are also involved in the detoxification of hydroxylamines, like sulfamethoxazole hydroxylamine (SMX-HA) [86]. All of these evidences together suggest that mARC has a strong role in the metabolic detoxification. Therefore, one of the mARC physiological roles could be avoiding the accumulation of mutagenic substances in the cell. Interestingly, in this sense, mARC has been found down-regulated in colon tumors [87].

Some NHC prodrugs are also mARC substrates; prodrugs are compounds that after their administration are metabolized to the pharmacological active drug [88]. Amidines are functional groups in many drugs that are used for the treatment of several diseases [89]. However, amidines have the inconveniences that are poorly absorbed by gastrointestinal tracts because they are easily protonated due to their strong basicity [90]. This problem can be solved by using the prodrug strategy. The amidoxime prodrug improves the bioavailability of the amidine moiety, since the electronegative charges resulting from oxygenation prevent the protonation of these compounds. Until now, nearly all of the amidoxime prodrugs tested have been activated by the human ARCO system [91]. In this sense, some examples of NHC prodrugs converted to its active form by human ARCO system are: Ximelagatran, an oral drug for direct thrombin inhibition [92,93], Mesupron, an urokinase inhibitor [94], 2,4,6-trimethylacetophenone oxime an anti-inflammatory agent, N-hydroxyamidinohydrazones (guanoxabenz), and amitriptyline-N-oxide antidepressant agents [95].

Recently, hydroxamic acids have also been proposed as substrates of human and porcine mARC [96]. Hydroxamic acids show a variety of pharmacological activities and are often used as prodrugs. In comparison with other known substrates of mARC (e.g., amidoxime), the conversion rates measured for the hydroxamic acids were slower, thereby reflecting the low metabolic stability and oral bioavailability of distinct hydroxamic acids [96].

As previously commented, there are usually two mARC members per genome (mARC1 and mARC2). Interestingly, some NHC are solely reduced by only one mARC member [95]. These data indicate that there might be some key structural differences in the mARC catalytic centers that would allow for each mARC member to participate in different metabolic reactions. That is the case of trimethylamine N-oxide, a molecule derived from dietary products containing compounds, such as betaine, L-carnitine, phosphatidylcholine, and choline. Where only mARC1 and not mARC2 reduces trimethylamine N-oxide to trimethylamine [97]. Interestingly, elevated levels of trimethylamine N-oxide in plasma correlate with an elevated cardiovascular disease risk [98]. These findings indicate that mARC might contribute to the prevention of cardiovascular diseases [97].

## 6. The Nitrite Reduction Capacity of mARC

Nitric oxide (NO) is an important gasotransmitter that is considered as a ubiquitous signaling molecule that is involved in many different biological processes from bacteria to humans [99]. Two pathways, the oxidative and the reductive, can be distinguished for NO production [100]. The oxidative pathway, catalyzed by NO synthase enzyme, involves NO production from L-arginine, in which N4-hydroxy-l-arginine (NOHA) is an intermediate. The human mARC enzymes have also been related to NO oxidative pathways, since they are able to catalyze, at least in vitro, the reduction of NOHA to arginine [101]. About the reductive NO production a variety of enzymes has been reported to reduce nitrite to NO, such as cytoglobin, deoxyhemoglobin, myoglobin [102,103], neuroglobin [104], cytochrome c [105], a plasma membrane-bound nitrite:NO reductase [106], the mitochondrial electron transport chain [100], as well as several Moco enzymes, such as XOR, NR, AO, and SO [45,107,108]. However, a new actor in the NO reductive pathway involving the mARC enzyme has been recently reported. In this sense, the human mARCs were the first one shown to catalyze the reduction of nitrite to NO using Cytb5-1 and Cytb5-R as partners [109]. Interestingly, the composition of this mini electron transport chain looks like the domain composition of plant NR enzyme. In this sense, the heme and the reductase domains of NR [110] have a remarkable similarity to Cytb5-1 and Cytb5-R [80]. In relation to this observation, plant mARC was able to synthesize NO from nitrite, but using NR as donor of the NADH electrons instead of Cytb5-1 and Cytb5-R. This new plant NR-dependent mARC activity was termed NO Forming Nitrite Reductase (NOFNiR) [80]. This NO synthesis occurs in the cytosol and it is strictly dependent on the NR diaphorase activity but independent of the NR Moco domain [80]. This NOFNiR activity can catalyze NO production from nitrite, even in the presence of millimolar concentrations of nitrate, which is known to strongly inhibit the NO production from NR [111]. These findings suggest that plant NR­mARC form an efficient machinery to synthesize NO under physiological conditions, aerobiosis, and in the presence of both nitrate and nitrite. In consequence, it is proposed that NR-mARC should have a role in plant/algal biology by modulating the cellular levels of NO [45].

### The mARC Reduction Mechanism of NHC Versus Nitrite

**Figure 4:**
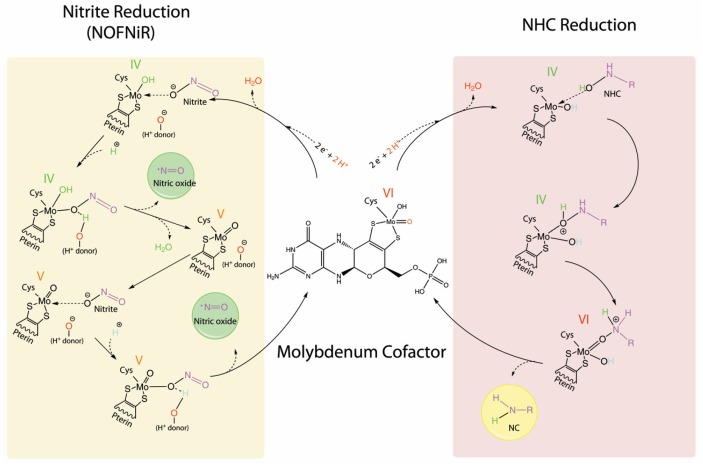
The mechanism of nitrite and N-hydroxylated (NHC) reduction catalysed by mARC. Shadowed in yellow the mechanism proposed for the nitrite reduction and shadowed in pink the mechanism that was proposed for the NHC reduction. For simplicity the full Moco structure is only represented in the first step, in the remaining steps the pyranopterin motif is mainly omitted (pterin) and only the dithiolate moiety is shown. The Mo Redox states are shown in colored roman numbers. Some atoms are shown in color to facilitate tracking their fate. In the NHC structure R means any compound.

As mentioned before, mARC can catalyze both NHC and nitrite reduction. However, the mechanistic details for these reductions are largely unknown. Based on recent discoveries, hypothetical mechanisms will be proposed and compared here. All known Moco enzymes are able to catalyze the nitrite reduction to NO; therefore, the core chemistry behind this reaction should not be deeply affected by the structural differences between the Mo center characteristics of each Moco enzyme. In this way, the different amino acid residues that are present in the active site of each enzyme would just modulate the nitrite binding and the catalytic efficiency. Thus, the mechanism that is proposed here for mARC nitrite reduction would be also applicable to the rest of the Moco enzymes. The mARC oxidized form possesses Mo (VI) coordinated by: one terminal oxo ligand, one hydroxyl group, a pyranopterin dithiolene, and a cysteine of the protein (Figure 4, center). Moco enzymes are known to redox cycle between Mo (VI) and a two-electron reduced Mo (IV). The nitrite reduction to NO should take place in the mARC Mo center, because the mutation of the human mARC C273, which coordinates de Mo center, creates an inactive tri­oxo Mo center that abolishes the NO formation [109]. The reduction of nitrite to NO is a one-electron step. In bioinorganic catalysis, the one-electron catalytic mechanism is widely known for first row transition metals but is poorly understood for pyranopterin Mo enzymes. First, the Mo (VI) center is reduced by NADH to Mo (IV) (Figure 4, left). The nitrite binding should occur after Mo reduction and through one of its oxygen atoms [112]. For the NO formation, the Mo center must promote one N­O bond cleavage, a step that is suggested to be triggered by a protonation event because it is greatly accelerated under acidic conditions [109]. Therefore, it was proposed that once the Mo(IV)­O­N­O complex is formed, the reaction proceeds with the protonation of the nitrite oxygen atom that is bound to the Mo, at the expense of a neighboring protonated residue (H^+^ donor in Figure 4), forming a ternary complex [113]. However, the residues responsible for this protonation have not been yet identified. The protonation step would produce the electron transfer from the reduced Mo to the now protonated nitrite, causing the N­OH bond homolysis, the subsequent NO, and one water molecule release and the Mo (IV) oxidation to Mo (V). In agreement with that, one­electron oxidized paramagnetic Mo (V) has been detected when the mARC enzyme was exposed to nitrite by electron paramagnetic resonance (EPR) [114]. The EPR signal showed evidence that Mo (V) is a mono-oxo species in which a single unpaired electron occupies a Mo redox orbital [114].

At this stage, one NO molecule is already formed. However, because the Mo center reduction is a two-electrons process, another nitrite molecule could be reduced. This reaction is suggested to proceed with the binding of another nitrite molecule. To generate a good leaving group, the consumption of one more proton is proposed and another cycle releases one more NO molecule, leaving the Mo into its original oxidation state (VI), prepared to start another catalytic cycle [113]. This mechanism might be applied to all Mo enzymes, as long as the reduced Mo center had an appropriate and accessible coordination position to bind nitrite.

The reduction of NHC is a two-electrons reduction (Figure 4, right). The two-electrons catalytic transformation mediated by Moco enzymes are coupled to the transfer of oxygen from the substrate to the reduced Mo center [115]. The NHC reduction should also take place in the mARC Mo center, because the mutation of the crARC C252 abolishes Moco binding and NHC reduction [65]. Consequently, we propose that the first step of the NHC reduction, the Mo (VI) reduction by NADH to Mo (IV), would be very similar to the nitrite reduction. However, once Mo (IV) is formed, a protonation event would not be needed, since the NHC oxygen has already bound hydrogen, forming a binary complex instead of ternary as in nitrite reduction. For the N­OH bond homolysis, the hydroxyl group would transfer a proton to nitrogen, leaving an oxo group that was bound to Mo. This will trigger the break of the N­OH bond, the transfer of the oxo group to the Mo center, and the N-compound (NC) liberation. This would leave Mo oxidized to Mo (VI) without an intermediate Mo (V) as in the nitrite reduction, and Mo (VI) coordinated to an oxo and a hydroxyl group that was ready to start another catalytic cycle. Nevertheless, future experiments would be required to verify these hypothetical mechanisms.

## 7. Are There Other mARC Substrates or Partners Still Uncovered?

Several studies have shown a close connection between mARC, lipogenesis, and diabetes. The mARC mutation has a significant effect on the fatty acid composition [93] and impairs the lipid synthesis in adipocytes [116]. The NHC reduction activity is high in the adipose tissue of rodents [117]. The phenotype of the mARC knockout mouse is characterized by a decreased fat level and increased lean body mass [91]. In mice, another study has shown that the expression and activity of the mARC system is affected by fasting and a high fat diet [95]. The mARC enzyme is induced under adipogenic conditions, and its expression is up-regulated in type 2 diabetes [118]. A study has described that certain structural variations of mARC might be related with diabetes [119].

These data indicate that mARC may be involved in lipogenesis and/or diabetes. However, how mARC regulates these processes and which ones are its partners or substrates still remain unsolved. In relation to this observation, we would like to propose two hypotheses. First, as mentioned, mARC is involved in the NO synthesis, and interestingly it has been shown that NO can interact with fatty acids to generate nitro-fatty acids, which have been identified as important signaling mediators with anti-inflammatory and antioxidant properties in animal and plant systems [120]. Therefore, we propose that mARC could be involved through its NO production capacity in regulating the levels of nitro-fatty acids and indirectly the lipogenesis by controlling the amount of free fatty acids. Second, Cytb5 plus Cytb5-R participates in several metabolic conversions, and interestingly, in coalition with a desaturase enzyme as partners, in the elongation and desaturation of fatty acids and cholesterol [121]. As Cytb5 plus Cytb5-R are mARC partners, we propose that, in some way, by kidnapping the partners or competing for the same substrate, mARC might regulate the lipogenesis. Future experiments are needed to substantiate the viability of these two hypotheses.

## 8. The Structural Modeling of a Eukaryotic MOSC Protein

**Figure 5:**
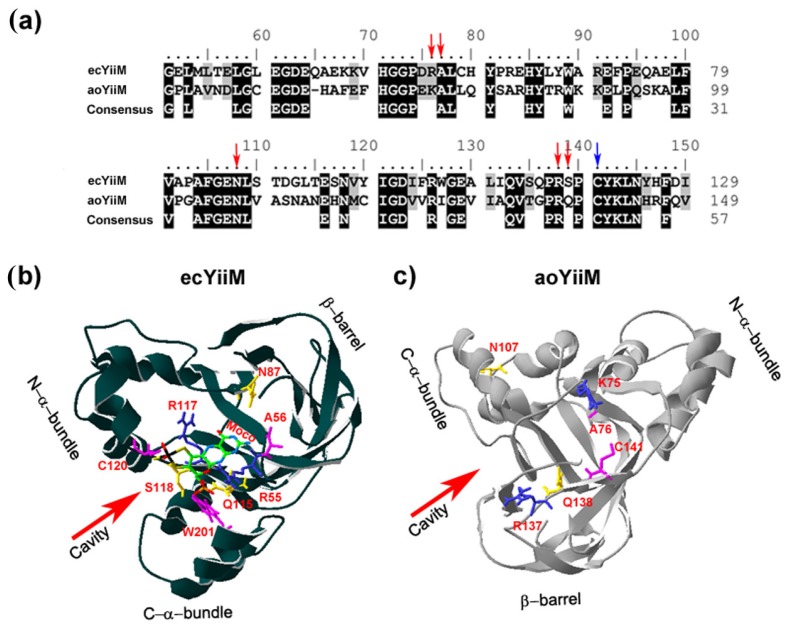
The in silico structure prediction of a eukaryotic MOSC member. (**a**) Sequence alignment of Escherichia coli YiiM (ecYiiM) (NP_418346) and Aspergillus oryzae YiiM (aoYiiM) (OAory_01080290). The consensus sequences have been calculated with a threshold of 75% with the BioEdit v.7.0.9 program. Highly conserved amino acids are shown in black background and moderately conserved amino acids in grey background. With blue arrowheads it is shown the invariant cysteine residue (C120 in ecYiiM and C141 in aoYiiM); (**b**) The secondary structure of ecYiiM (PDB ID: 5YHI) and its putative Moco binding site in its cavity are shown. The amino acid residues highlighted in red are the ones described in [122] as interacting with the Moco structure. In the Moco structure, the colors of the atoms are: C (green), N (Blue), O (Red), S (Yellow), P (Orange), and Mo (Black). The colors of the amino acids side chain are: positive charges amino acids (blue), hydrophobic ones and cysteine (pink) and polar amino acids (yellow); (**c**) Predicted in silico structure of aoYiiM. The protein used as model was ecYiiM. The identity between both sequences is 29.8%. The six highlighted amino acid residues are the ones marked in (**a**) with red arrowheads (K75, A76, N107, R137, Q138) and blue arrowheads (C141). The aoYiiM structure was obtained with the Swiss-PdbViewer Program of tertiary structures and three-dimensional alignments of already crystallized structures online version http://spdbv.vital-it.ch/ Swiss-Model.

The mARC proteins belonging to the MOSC family in eukaryotes have an unknown structure. However, in prokaryotes, it has recently been reported the first three-dimensional (3D) structure of Escherichia coli YiiM (ecYiiM), a MOSC family member [122]. ecYiiM has been involved in the reduction of the mutagenic HAP [81]. A eukaryotic enzyme that is closely related to ecYiiM is Aspergillus oryzae YiiM (aoYiiM). The sequence conservation indicates that aoYiiM belongs to the MOSC family, although its function has not been characterized yet (Figure 5a). The ecYiiM structure folds into a triangular shape with ten β-strands and six α-helices, which can be divided into three distinct structural domains: a β-barrel, an N-terminal α-helix bundle (N-α-bundle) and a C-terminal α-helix 1 (C-α-bundle) (Figure 5b). The β-barrel domain is decorated with the N-α-bundle and the C-α-bundle, each of which forms one vertex of the YiiM triangle. In the middle of its triangular architecture, ecYiiM possesses a cavity. Interestingly, the surface of this cavity has a positive electrostatic potential, presumably to hold a negatively charged molecule, such as Moco. The residues of this cavity are highly conserved in MOSC proteins, suggesting a critical role of this cavity in the enzymatic function of this family. This cavity holds the invariant cysteine residue (C120, in Figure 5b), which is absolutely conserved in all MOSC proteins [70]. Therefore, MOSC proteins could employ this invariant cysteine as a Moco-conjugating residue or a catalytic essential residue [122]. The Moco electron density was not visible in ecYiiM three-dimensional (3D) structure. Interestingly, a phosphate ion was otherwise found in the cavity near the invariant cysteine residue. Since Moco contains a phosphate group at one end, by in silico docking studies, this phosphate ion was used as a guide to locate the Moco molecule in the YiiM structure [122]. In Figure 5b, several aminoacid residues within the proximity of Moco are highlighted and how the Moco molecule fills the ecYiiM cavity is shown. This prediction shows that Moco fits in this cavity quite well and it is stabilized by multiple hydrogen bonds with the side chains of cavity residues [122].

We have modeled the aoYiiM 3D structure based on ecYiiM. The aoYiiM structure that was obtained looks very similar to ecYiiM (Figure 5c). It can be observed that six of the conserved amino acids shown in Figure 5a (K75, A76, N107, R137, Q138, C141) are forming a cavity in the center of the aoYiiM triangle. The side chain of the invariant cysteine residue (C141) faces this cavity. We propose that this could be the place where Moco is bound. To verify the reliability of this model, more structural studies of eukaryotic MOSC members will be required to reveal the exact Moco binding site, which is essential to reveal its catalytic mechanism.

## 9. Notes Added in Proof

While this article was under review, the first crystal structure of a eukaryotic mARC was resolved, the human mARC1 [123].
